# Deducing the Kinetics of Protein Synthesis *In Vivo* from the Transition Rates Measured *In Vitro*

**DOI:** 10.1371/journal.pcbi.1003909

**Published:** 2014-10-30

**Authors:** Sophia Rudorf, Michael Thommen, Marina V. Rodnina, Reinhard Lipowsky

**Affiliations:** 1 Theory and Bio-Systems, Max Planck Institute of Colloids and Interfaces, Potsdam, Germany; 2 Physical Biochemistry, Max Planck Institute for Biophysical Chemistry, Göttingen, Germany; University of Missouri, United States of America

## Abstract

The molecular machinery of life relies on complex multistep processes that involve numerous individual transitions, such as molecular association and dissociation steps, chemical reactions, and mechanical movements. The corresponding transition rates can be typically measured *in vitro* but not *in vivo*. Here, we develop a general method to deduce the *in-vivo* rates from their *in-vitro* values. The method has two basic components. First, we introduce the kinetic distance, a new concept by which we can quantitatively compare the kinetics of a multistep process in different environments. The kinetic distance depends logarithmically on the transition rates and can be interpreted in terms of the underlying free energy barriers. Second, we minimize the kinetic distance between the *in-vitro* and the *in-vivo* process, imposing the constraint that the deduced rates reproduce a known global property such as the overall *in-vivo* speed. In order to demonstrate the predictive power of our method, we apply it to protein synthesis by ribosomes, a key process of gene expression. We describe the latter process by a codon-specific Markov model with three reaction pathways, corresponding to the initial binding of cognate, near-cognate, and non-cognate tRNA, for which we determine all individual transition rates *in vitro*. We then predict the *in-vivo* rates by the constrained minimization procedure and validate these rates by three independent sets of *in-vivo* data, obtained for codon-dependent translation speeds, codon-specific translation dynamics, and missense error frequencies. In all cases, we find good agreement between theory and experiment without adjusting any fit parameter. The deduced *in-vivo* rates lead to smaller error frequencies than the known *in-vitro* rates, primarily by an improved initial selection of tRNA. The method introduced here is relatively simple from a computational point of view and can be applied to any biomolecular process, for which we have detailed information about the *in-vitro* kinetics.

## Author Summary

The proverb ‘life is motion’ also applies to the molecular scale. Indeed, if we looked into any living cell with molecular resolution, we would observe a large variety of highly dynamic processes. One particularly striking aspect of these dynamics is that all macromolecules within the cell are continuously synthesized, modified, and degraded by complex biomolecular machines. These ‘nanorobots’ follow intricate reaction pathways that form networks of molecular transitions or transformation steps. Each of these steps is stochastic and takes, on average, a certain amount of time. A fundamentally important question is how these individual step times or the corresponding transition rates determine the overall speed of the process in the cell. This question is difficult to answer, however, because the step times can only be measured *in vitro* but not *in vivo*. Here, we develop a general computational method by which one can deduce the individual step times *in vivo* from their *in-vitro* values. In order to demonstrate the predictive power of our method, we apply it to protein synthesis by ribosomes, a key process of gene expression, and validate the deduced step times by three independent sets of *in-vivo* data.

## Introduction

Life is based on the continuous synthesis, modification, and degradation of proteins and other macromolecules. These processes are performed by complex biomolecular machines that bind their ligands and transform them into product molecules. Examples are provided by the transcription of DNA by RNA polymerases, the translation of mRNA by ribosomes, or the degradation of proteins by proteasomes. Each of these processes involves several steps: the binding of the ligand molecules, chemical reactions catalyzed at the active sites, as well as specific conformational changes and directed mechanical movements of parts of the molecular machinery. In principle, the kinetics of such multistep processes can be understood in terms of the individual transitions and the associated transition rates, a well-established approach both for enzyme kinetics [1]–[3] and for free energy transduction by molecular motors [4], [5]. In practice, the values of the individual transition rates can be typically measured *in vitro* but not *in vivo*, and the *in-vitro* rates depend on the composition of the buffer. Because the cytosol represents a rather complex buffer, it is difficult to assess whether a certain *in-vitro* assay provides a reliable description of the process *in vivo*. One important tool that is missing for such an assessment is a simple measure by which we can quantitatively compare the kinetics of a multistep process in different environments.

Here, we develop a general method that provides such a measure and allows the deduction of the *in-vivo* rates from their *in-vitro* values. Our method has two basic components. First, we introduce the ‘kinetic distance’, i.e., a distance metric for the kinetics, by which we can describe the similarity or dissimilarity of multistep processes *in vitro* and *in vivo* in a quantitative manner. The kinetic distance depends logarithmically on the rates and has an intuitive interpretation in terms of the associated free energy barriers. Second, we minimize the kinetic distance between the *in-vitro* and *in-vivo* processes, imposing the constraint that the deduced rates reproduce a known global property such as the overall *in-vivo* speed. Computationally, this constraint defines a hypersurface in the multi-dimensional space of transition rates. In order to demonstrate the predictive power of our method, we apply it to the elongation cycle of protein synthesis, a key process of gene expression.

In all living cells, proteins are synthesized by ribosomes, which translate the codon sequences of mRNA into peptide chains of proteins. During the elongation cycle of this process, the ribosome translates one codon after another by binding a ternary complex consisting of aminoacyl-tRNA (aa-tRNA), elongation factor Tu (EF-Tu) and GTP. The amino acid is transferred from the tRNA to the nascent peptide chain, and the ribosome moves to the next codon with the help of elongation factor G (EF-G) allowing for the next elongation cycle. Translation elongation involves several individual states with rapid transitions between them [6], [7]. The different states have been studied by a variety of experimental techniques: chemical probing methods [8], pre-steady state kinetics [9]–[15], electron microscopy [16]–[19], X-ray crystallography [20]–[22], and single molecule methods [23], [24]. The kinetic measurements *in vitro* provided values for the individual transition rates but, so far, it has not been possible to measure the corresponding rates in the cell.

**Figure 1:**
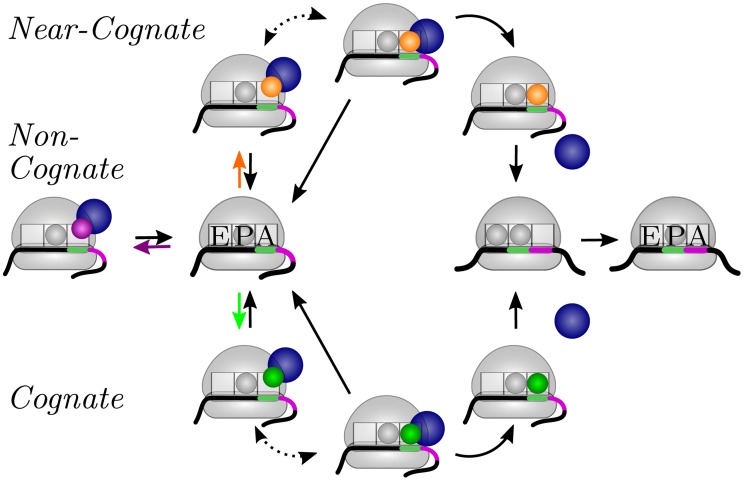
Elongation cycle of a ribosome (gray dome) translating an mRNA (black-green-purple line). Aminoacyl-tRNA (small gray, green, purple, or orange sphere) is delivered to the ribosome in a ternary complex with the elongation factor EF-Tu (larger blue sphere) and GTP (not shown). In addition to the initial binding site, the ribosome has three tRNA binding sites, the A, P, and E sites. The elongation cycle of translation starts when the A site of the ribosome has arrived at a new codon (green) of the mRNA. The ribosome then binds a ternary complex with a tRNA that may be cognate, near-cognate, or non-cognate to this codon. As a consequence, the elongation cycle exhibits three different branches corresponding to three different reaction pathways: (left) A non-cognate ternary complex is again released from the initial binding site of the ribosome; (top) A near-cognate ternary complex is usually rejected but is very rarely used to elongate the peptide chain; and (bottom) A cognate ternary complex may also be rejected but is typically used for elongation of the peptide chain. The two dotted arrows correspond to additional intermediate states and transitions as explained in more detail in Fig. 3 below.

The different states and transitions of the elongation cycle are schematically shown in Fig. 1. When the ribosome dwells at a certain codon and binds a ternary complex, the tRNA within this complex can be cognate, near-cognate, or non-cognate to the codon, which implies that the elongation cycle contains three different reaction pathways corresponding to the three branches in Fig. 1. During each round of elongation, the ribosome typically explores all three pathways in order to select a cognate tRNA and to reject the near-cognate and non-cognate ones. The individual rates of these pathways were measured *in vitro* at 20°C and/or 37°C using the ribosomes and translation factors from *Escherichia coli* [6], [13], [25]. Here, we combine these results with new data on the overall elongation rates *in vitro* to first derive a complete set of individual *in-vitro* rates at both temperatures. We then minimize the kinetic distance between the *in-vitro* and *in-vivo* processes, taking into account two known properties of the *in-vivo* process: the overall elongation rates [26], [27] and the tRNA concentrations [28], both of which have been measured in *E. coli* for different growth conditions.

The *in-vivo* rates of the elongation cycle obtained in this way are then validated by three independent sets of *in-vivo* data [29]–[31]. First, we compute codon-specific elongation rates and show that these rates correlate well with relative translation rates as obtained experimentally by [29]. Second, we predict the time-dependent incorporation of radioactively labeled amino acids into proteins as studied *in vivo* by [30]. The time course of synthesis obtained theoretically is in excellent agreement with the experimental data. Third, using the same *in-vivo* rates, we also compute the missense error frequency and obtain good agreement with the experimental results of [31]. In all three cases, our computations do not involve any fit parameter and, thus, directly validate the derived set of *in-vivo* rates.

## Results

### Distance between *in-vitro* and *in-vivo* kinetics

In order to introduce a quantitative measure for the (dis)similarity of the *in-vivo* and *in-vitro* kinetics, we consider a generic multistep process within the cell and first focus on one of the individual transitions from state 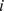 to state 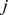. The corresponding transition rates have the values 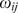 and 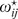 for a certain *in-vitro* assay and for specific *in-vivo* growth conditions, respectively. Instead of the rates, we can equally well consider the associated transition times 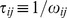 and 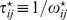. Thus, we require that the distance 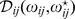 between the rates 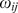 and 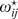 is equal to the distance 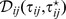 between the times 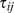 and 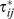, i.e., that
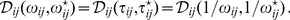
(1)


The *simplest* expression for 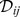 that fulfills this requirement is provided by
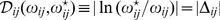
(2)with the logarithmic difference
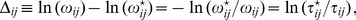
(3)between the *in-vitro* and the *in-vivo* value of the individual transition rate.

The single transition distance 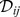 is dimensionless and does not involve any parameter apart from the two rates 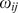 and 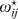. In addition, this distance satisfies the two scaling relations 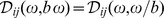 and 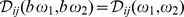 for any rescaling factor 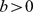. The first scaling relation implies that 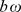 and 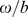 have the same distance from 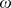, which agrees with our intuition. The second scaling relation implies that the distance 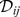 does not depend on the units used to measure the rates. For small deviations of 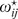 from 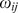, which are equivalent to small deviations of 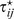 from 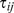, the distance 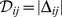 becomes asymptotically equal to both 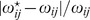 and 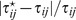.

**Figure 2:**
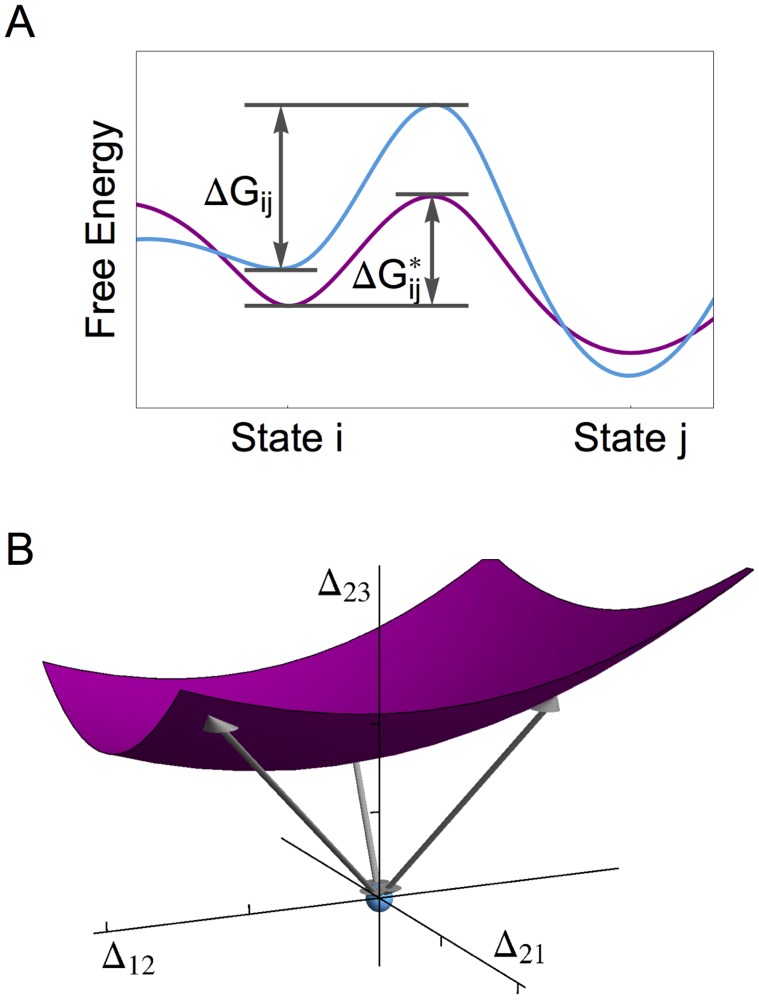
Kinetic distance based on the logarithmic differences 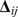 between the *in-vitro* and *in-vivo* rates. (A) *In-vitro* free energy barrier 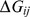 and *in-vivo* barrier 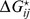 for the transition from state 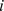 to state 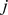. When expressed in units of 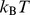, the single barrier shift 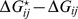 determines the logarithmic difference 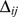, see Eq. 5; and (B) Three-dimensional section of the multi-dimensional barrier space with coordinates 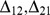, and 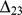. The origin of this space (light blue dot) corresponds to the *in-vitro* system. The surface (purple) represents a two-dimensional section of the hypersurface described by Eq. 8, corresponding to a fixed *in-vivo* value for the global kinetic quantity. Each point on this surface has a certain kinetic distance that is equal to the Euclidean distance of this point from the origin, as indicated by the three double arrows. The point with the *shortest* kinetic distance determines the predicted *in-vivo* rates 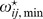.

The *in-vitro* rate 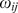 can be expressed in terms of the activation free energy or free energy barrier 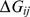 and the attempt frequency 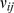 which leads to
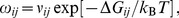
(4)where the thermal energy 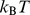 provides the basic free energy scale. When we combine this expression with the analogous expression for the *in-vivo* rate, the logarithmic difference between the two rates becomes



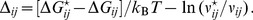
(5)Because the prefactors 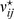 and 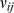 are expected to have the same order of magnitude, the second term 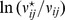 should usually be small compared to the first term which represents the shift of the free energy barrier between state 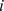 and state 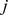, see Fig. 2A. Therefore, for each individual transition along one of the reaction pathways, the logarithmic difference 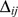 can be interpreted as the shift of the free energy barrier that governs the transition from state 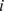 to state 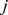. In the following, we will use the intuitive terminology ‘single barrier shift’ for the quantity 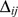. It should be noted, however, that, in spite of this terminology, changes in the attempt frequency as described by the term 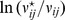 in Eq. 5 are included in the logarithmic difference 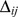 and, thus, will be taken into account in all our calculations.

Next, we consider all individual transitions along the reaction pathways of the multistep process and regard the associated *in-vivo* rates 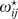 as unknown variables that can be visualized as the coordinates of a multi-dimensional space. These coordinates are somewhat impractical, however, because they are restricted to positive values. In order to eliminate this restriction, we perform a coordinate transformation from the *in-vivo* rates 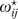 to the single barrier shifts 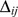, which can attain both positive and negative values. This coordinate transformation is highly nonlinear but invertible with the inverse transformation given by 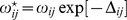.

The overall distance 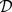 between the *in-vitro* and the *in-vivo* kinetics is now defined by
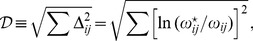
(6)where the summation under the square root runs over all individual transitions along the reaction pathways. As illustrated in Fig. 2B, the distance 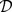 represents the Euclidean distance within the multi-dimensional space defined by the single barrier shifts 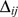. Therefore, the distance 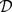 provides a genuine metric in the mathematical sense, which implies that it satisfies the triangle inequality if we compare three different *in-vitro* and/or *in-vivo* conditions.

If all *in-vivo* rates are identical to their *in-vitro* values, apart from a single one, 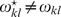, Eq. 6 for the kinetic distance 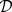 reduces to Eq. 1 for the single transition distance 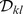. Because the choice of the two states 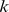 and 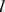 is arbitrary, this property of the kinetic distance applies to all individual transitions 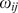 that enter in Eq. 6. The latter property represents, in fact, a general requirement for any meaningful definition of the kinetic distance. Therefore, if we considered the more general expression 
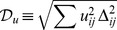
 with dimensionless weight factors 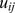, this requirement would imply that all weight factors must assume the unique values 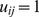 and that 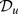 must be equal to the kinetic distance 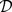 as given by Eq. 6.

If we consider two different *in-vitro* assays, say 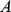 and 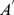, the corresponding transition rates 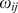 and 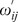 will, in general, be different and define two sets of single barrier shifts *via* 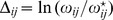 and
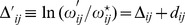
(7)with the logarithmic differences 
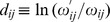
. The latter quantities determine the kinetic distance 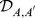 between the two *in-vitro* assays, which is given by 
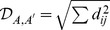
. The two sets of barrier shifts, 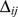 and 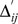, provide two different coordinates for the multi-dimensional barrier space. Because of the linear relations as given by Eq. 7, the primed coordinates are obtained from the unprimed ones by shifting the latter coordinates by the logarithmic differences 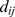. Therefore, the transformation from the unprimed to the primed coordinates, corresponding to a change from assay A to assay A′, represents a Euclidean translation of the coordinate system, which preserves the shape of any geometric object within the multi-dimensional barrier space.

### Constrained minimization of the kinetic distance

Next, we combine the kinetic distance as given by Eq. 6 with a minimization procedure to predict the unknown *in-vivo* rates from their known *in-vitro* values. Even though the rates of the individual transitions are difficult to study in the cell, one can usually measure some quantity that characterizes the overall kinetics of the intracellular process. One such quantity is provided by the average speed of the process. Any such global kinetic quantity, 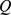, depends on the individual transition rates, 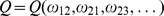. The *in-vivo* values 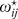 of the individual transition rates must reproduce the experimentally measured value 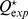 of the global quantity. This requirement implies the equation
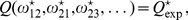
(8)which represents a constraint on the unknown *in-vivo* values 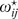. This constraint can be expressed in terms of the single barrier shifts 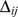 using the inverse coordinate transformation 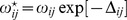 with the known *in-vitro* values 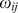. As a result, the constraint in Eq. 8 defines a hypersurface in the multi-dimensional barrier space as illustrated in Fig. 2B. Each point on this hypersurface is compatible with the measured value 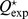 of the global kinetic quantity. In addition, the Euclidean distance of such a point from the origin is equal to the kinetic distance 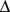 between the (unknown) *in-vivo* and the (known) *in-vitro* values of the transition rates. Our prediction for the *in-vivo* values 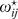 is then obtained by minimizing this kinetic distance, i.e, by the point on the hypersurface that has the *shortest* distance from the origin. For clarity, the coordinate values of this point will be denoted by 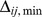 in order to distinguish these values from the variable coordinates 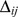.

Our approach involves the following assumptions. First, we make the usual assumption that the states of the biomolecular system that have been identified *in vitro* are also present *in vivo*. The molecular conformations of the corresponding *in-vitro* and *in-vivo* states are expected to be somewhat different when viewed with atomic resolution, but the gross features of these conformations should be similar, in particular when the *in-vitro* assay is functional and has been optimized. It is then plausible to assume that the *in-vitro* and *in-vivo* values of the individual transition rates do not differ by many orders of magnitude, which implies that the point in the multi-dimensional barrier space that represents the true *in-vivo* rates is located ‘in the neighborhood’ of the origin of this space and, thus, characterized by a ‘small’ kinetic distance 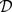. If the kinetic distance satisfied 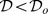, the true *in-vivo* point would be located within a sphere of radius 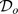 around the origin. The smallest sphere that is compatible with the *in-vivo* constraint as given by Eq. 8 is the one that touches the hypersurface depicted in Fig. 2B, and the radius 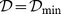 of this sphere is equal to the Euclidean distance of the hypersurface from the origin of the Δ*_ij_*-coordinates. The associated contact point between 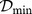-sphere and hypersurface represents the predicted *in-vivo* point, and its coordinate values 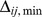 lead to the predicted *in-vivo* rates 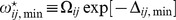 based on the known *in-vitro* rates 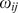.

For a general, nonlinear *in-vivo* constraint, the coordinate values 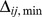 of the predicted *in-vivo* point will be different for different individual transitions. The minimization procedure then predicts different scale factors 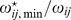 and, thus, different effects of the *in-vivo* environment on the individual transitions of the system. Such differences are indeed obtained when we apply our minimization approach to the kinetics of ribosomes as described in the next subsection. It is important to note that this approach leads to different scale factors even though the expression for the kinetic distance (Eq. 6) does not include any bias for one of the individual barrier shifts 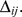 Therefore, the different scale factors 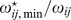 follow from the imposed *in-vivo* constraint (Eq. 8) alone and do not involve any additional assumptions or expectations about the *in-vivo* conditions.

The minimization procedure described above represents an extremum principle with constraints. Such principles have been successfully applied in many areas of science, in particular in the context of optimization problems. One important and useful feature of extremum principles is that they provide *global* solutions for *nonlinear* systems. Thus, in the present context, we would obtain a prediction for the *in-vivo* rates even if the *in-vitro* assay were rather different from the *in-vivo* conditions. Another advantage of extremum principles is that they typically lead to a *unique* solution without any additional assumptions (‘principle of least prejudice’). In some exceptional cases, one may find more than one solution, which then indicates that the system undergoes some kind of bifurcation. For the kinetics of ribosomes, see next subsection, we always found a unique solution and, thus, a unique set of predicted *in-vivo* rates.

The rates 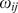 of the *in-vitro* assay are only known with a certain accuracy. As a consequence, the predicted *in-vivo* rates 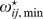 have some uncertainty as well. As explained in the *Methods* section, this uncertainty reflects both the accuracy of the measured *in-vitro* rates and the associated changes in the location of the predicted *in-vivo* point. Furthermore, the latter location will also depend, in general, on the rates of the chosen *in-vitro* assay. Indeed, the change from assay 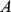 to assay 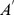 corresponds to a Euclidean translation of the coordinate system (Eq. 7) while the shape of the hypersurface (Eq. 8 and Fig. 2B) remains unchanged. These two properties imply that the distance of the hypersurface from the origin of the 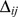-coordinates may differ from the distance of this surface from the origin of the 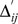-coordinates. Therefore, the validity of the predicted *in-vivo* rates 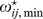 is difficult to assess *a priori*, but can be checked *a posteriori* in a self-consistent manner: we first deduce the unknown *in-vivo* rates from the known *in-vitro* rates *via* the minimization procedure and subsequently validate the deduced rates 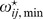 by calculating some other quantities that have been experimentally studied *in vivo*. In the next two subsections, we will apply this two-step procedure to the kinetics of ribosome elongation based on the *in-vitro* assay developed in [6], [25].

Our minimization procedure becomes computationally simpler if we have additional knowledge about some of the *in-vivo* values 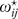 of the individual transition rates. If we knew one of these rates, e.g., 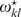, we would restrict our minimization procedure to the subspace with constant 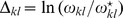. As a consequence, we would not vary the coordinate 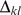 during the minimization and use the constant value of this coordinate in Eq. 6 for the kinetic distance 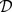. On the other hand, if we knew only that the *in-vivo* rate 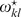 is located within the range 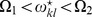, we would minimize the kinetic distance also with respect to 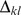 but within the subspace defined by 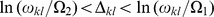. The latter procedure may lead to a boundary minimum, i.e., to a predicted *in-vivo* point that is located at the boundary of the considered subspace. Another simplification is obtained if the rates of two individual transitions, say from state 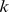 to state 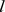 and from state 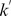 to state 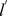, have the same values *in vitro* and *in vivo*, i.e., if 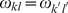 and 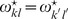. We will then reduce the multi-dimensional barrier space to the subspace with 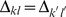, and the corresponding expression for the kinetic distance 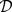 in Eq. 6 will now contain the term 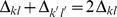 under the square root. The latter reduction will be used in the next subsection on the kinetics of ribosomes for which different individual transition rates have the same *in-vitro* values.

### Kinetics of ribosomes during protein synthesis

**Figure 3:**
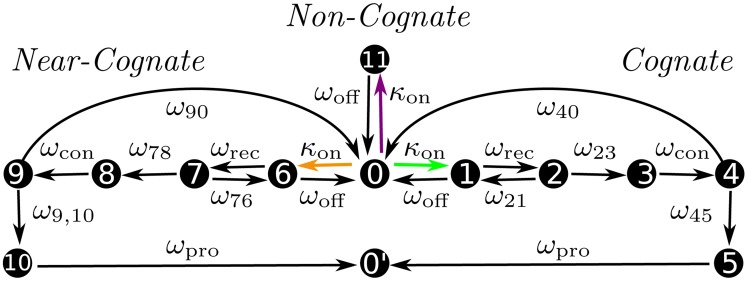
Codon-specific Markov process for translation elongation based on 12 ribosomal states for each codon 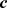. The elongation cycle starts in state 0 corresponding to a ribosome without any bound ternary complex. Initial binding of a cognate, near-cognate, or non-cognate ternary complex is indicated by the green, orange, and purple arrow, compare the color code in Fig. 4; the corresponding association rates are proportional to the association rate constant 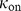 as in Eq. 9. The black arrows represent the individual transitions along the reaction pathways. All ternary complexes dissociate initially with the same dissociation rate 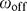. Likewise, cognate and near-cognate ternary complexes are governed by the same recognition rate 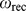, conformational rate 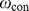, and processing rate 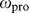. The kinetic distinction between the cognate and near-cognate branches arises from initial selection at the states 2 and 7 as well as from proofreading at the states 4 and 9.

Our quantitative description of the translation elongation cycle is based on the codon-specific Markov process displayed in Fig. 3. This process can visit, for each sense codon 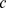, twelve ribosomal states, numbered from 0 to 11. After the ribosome has moved to the next sense codon, it dwells in state 0, until it binds a ternary complex with an elongator tRNA that may be cognate, near-cognate, or non-cognate to codon 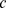.

**Figure 4:**
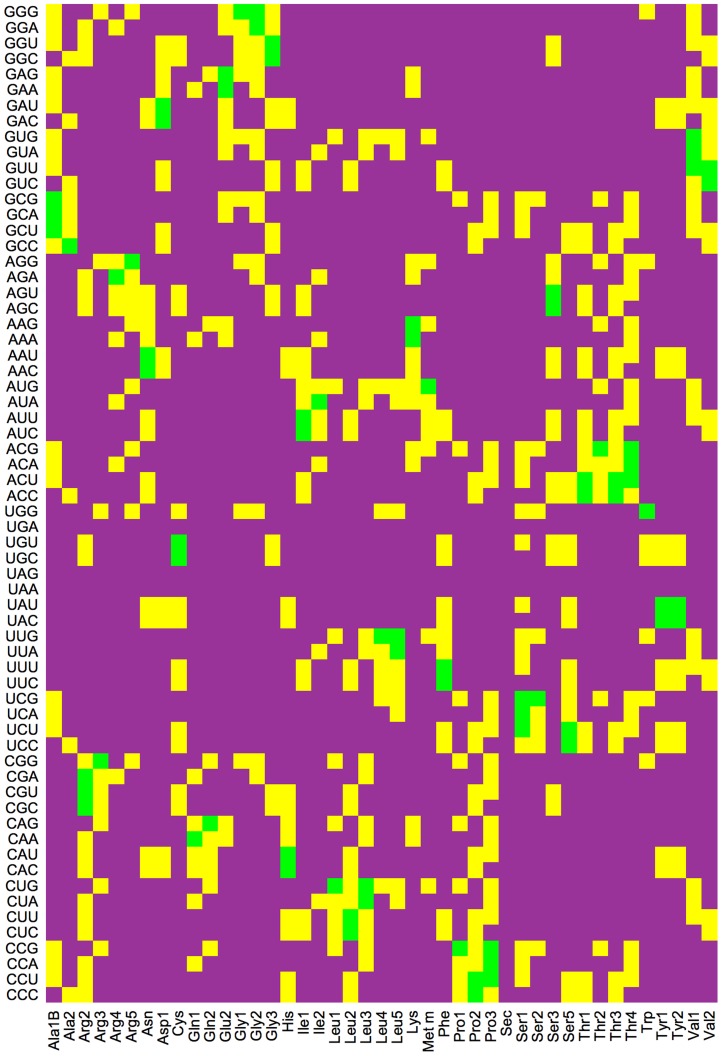
Decoding pattern arising from the cognate (green), near-cognate (yellow), and non-cognate (purple) relationships between all 61 sense codons and the 43 elongator tRNA species of *E. coli* as identified in Ref. [28]. For each tRNA species, the near-cognate codons differ from the cognate ones by a mismatch in one position of the codon-anticodon complex.

The genetic code involves 61 sense codons, which encode 20 proteinogenic amino acids and are decoded by a certain number of elongator tRNAs. The latter number depends on the organism but is always larger than 20 and smaller than 61 [32], [33]. For *E. coli*, 43 distinct species of elongator tRNA have been identified [28]. The corresponding codon-tRNA relationships can be visualized by the large matrix in Fig. 4 with 61 rows and 43 columns. As shown by the color code in this figure, each sense codon defines a *different* decomposition of the total set of tRNA species into three subsets of cognates, near-cognates, and non-cognates. The corresponding molar concentrations 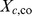, 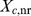, and 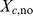 of cognate, near-cognate, and non-cognate ternary complexes determine the association rates
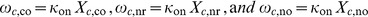
(9)for initial binding with the pseudo-first-order association rate constant 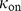. This constant is taken to be independent both of the codon and of the ternary complex as observed *in vitro* [10], [25]. The latter experiments also imply that all ternary complexes dissociate with the same rate 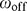 from the initial binding site and that the cognate and near-cognate ternary complexes have the same recognition rate 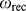.

After initial binding of a *non-cognate* ternary complex, this complex dissociates without visiting any other state, so that the ribosome returns back to state 0 with an empty initial binding site. Initial binding of a *cognate* ternary complex leads to state 1, from which the ternary complex can be released with rate 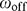 or can move into the A site to attain the codon recognition state 2 with rate 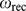. When the ternary complex is recognized as cognate, the ribosome undergoes a forward transition from state 2 to state 3, which corresponds to the combined process of GTPase activation of the cognate ternary complex and GTP hydrolysis, followed by the irreversible transition from state 3 to state 4, which describes phosphate release and conformational rearrangements of EF-Tu [6]. From state 4, the cognate ternary complex may either move to become fully accommodated into the A site via a transition from state 4 to state 5 or, with low probability, may be released from the A site via a transition from state 4 to state 0. After the cognate ternary complex has been fully accommodated, the ribosome/tRNA complex undergoes the final transition from state 5 into the empty state 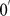 at the next codon 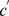. This transition describes the combined process of peptide bond formation and translocation, the corresponding processing rate is denoted by 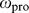.

Initial binding of a near-cognate ternary complex leads to state 6, from which the ternary complex can be released with rate 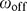 or move to the codon recognition state 7 with rate 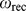. When the ternary complex is recognized as near-cognate, it is rejected and the ribosome undergoes a backward transition from state 7 to state 6, which provides the initial selection step during the decoding process. With low probability, the near-cognate ternary complex undergoes an irreversible transition from state 7 to state 8, corresponding to GTPase activation and GTP hydrolysis, as well as from state 8 to state 9, which describes phosphate release and conformational rearrangements of EF-Tu. From state 9, the near-cognate ternary complex is typically released again via a transition from state 9 to state 0, which provides the proofreading step during decoding. Very rarely, the near-cognate ternary complex is fully accommodated via a transition from state 9 to state 10. After a near-cognate tRNA has been fully accommodated, it is further processed via peptide bond formation and translocation and undergoes the transition from state 10 to state 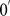 with rate 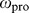.

Apart from the association rate constant 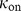, the kinetics of the elongation cycle then involves 12 different transition rates 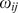 for the 17 transitions along the cognate, near-cognate, and non-cognate branches of the Markov process. All of these transition rates have been determined *in vitro* for the high-fidelity buffer developed in [12], [25], [34]. The corresponding *in-vitro* values are reported in Table 1. A few individual rates were measured at both 20 and 37°C whereas most of these rates were obtained either at 20 or at 37°C. We used a variety of computational methods to obtain complete and consistent sets of individual rates at both temperatures as described in the *Methods* section. In addition, we measured the overall elongation rate 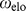 *in vitro* for a model protein, 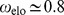 aa/s for 20°C and 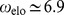 aa/s for 37°C (Supporting Figure S1). As explained in the *Methods* section (Eq. 22), the measured value of the overall elongation rate 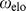 was then used to compute, for both temperatures, the *in-vitro* value 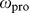 of the processing rate. The results of these computations are included in Table 1.

**Table 1:** *In-vitro* rates of ribosomal transitions.

Rates	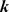 -not.	20°C	37°C	Units
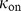	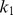	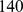 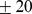	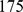 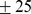	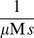
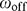	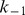	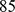 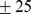	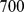 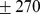	1/s
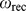	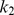	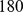 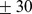	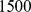 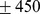	1/s
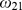	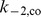	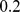 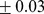	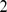 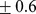	1/s
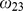	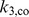	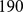 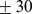	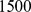 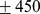	1/s
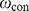	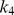	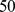	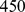	1/s
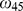	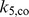	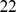 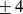	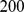 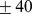	1/s
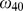	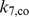	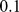	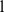	1/s
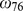	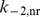	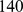 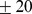	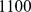 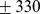	1/s
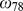	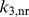	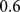 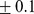	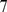 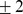	1/s
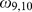	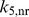	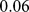 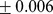	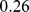 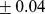	1/s
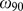	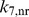	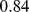 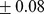	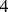 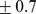	1/s
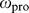		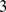 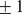	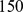 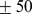	1/s
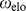		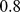 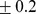	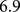 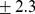	aa/s

Apart from the processing rate 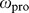, all individual rates and the overall elongation rate 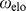 have been measured *in vitro* at 20°C and/or 37°C. The processing rate 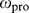 was calculated from the overall elongation rate 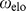 via Eq. 22 in the *Methods* section. The column ‘k-not.’ provides the notation for the transition rates as used in Ref. [6].

To predict the unknown *in-vivo* rates 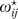 from the known *in-vitro* rates 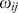, we consider the multi-dimensional space of single barrier shifts as described by the coordinates 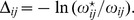 Because several transition rates of the Markov process considered here have the same values (Fig. 3), we use the resulting equalities for the associated coordinates as given by 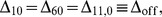 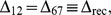 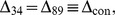 and 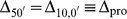 to reduce the 17-dimensional barrier space to a 12-dimensional subspace and restrict the minimization procedure of the kinetic distance to this subspace. After this reduction, the latter distance has the explicit form
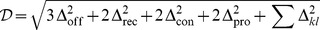
(10)where the sum 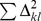 contains all the remaining transition rates of the Markov process in Fig. 3.

**Figure 5:**
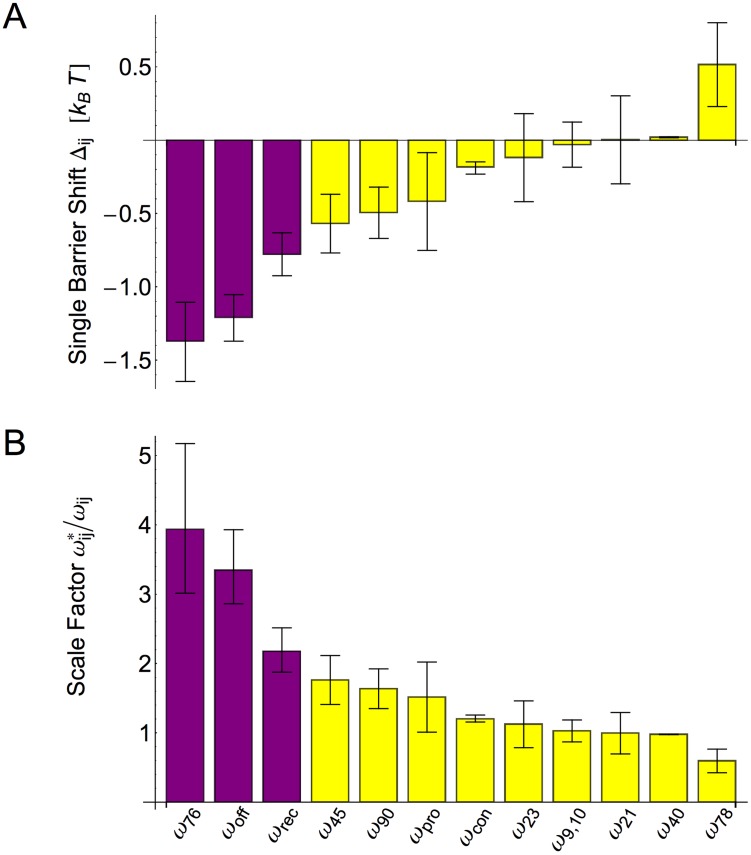
Quantitative comparison between the *in-vitro* kinetics of translation elongation at 37°C and the *in-vivo* kinetics as deduced for the growth condition of 2.5 dbl/h. (A) Single barrier shifts 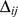 for the individual transition rates, see Eq. 2 and Fig. 2; and (B) Scale factors 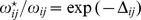 for all individual transitions of the ribosomes. A barrier shift 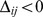 implies that the *in-vivo* rate 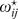 is increased compared to the *in-vitro* rate 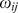.

Because the *in-vivo* experiments are typically performed at 37°C, we use the *in-vitro* values 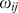 for the same temperature, see Table 1. Furthermore, we take into account the known *in-vivo* values of the overall elongation rate 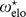 at different growth conditions [26], [27]. For each growth condition, the constraint in Eq. 8 now has the explicit form as given by Eq. 23 in the *Methods* section. As a result of the constrained minimization procedure, we find the *in-vivo* rates 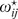 as given in Table 2 and the single barrier shifts 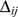 displayed in Fig. 5A, where we have again omitted the subscript ‘min’ for notational simplicity.

**Table 2:** *In-vivo* rates of ribosomal transitions.

Rates	*E. coli* growth rates [dbl/h]				RSD	Units
	0.7	1.07	1.6	2.5		
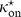	94	94	94	94	0.1	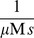
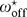	1400	1700	2100	2300	0.4	1/s
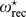	2100	2500	3000	3300	0.3	1/s
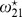	2	2	2	2	0.3	1/s
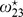	1600	1600	1700	1700	0.3	1/s
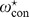	490	500	530	540		1/s
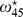	270	300	340	350	0.2	1/s
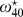	1	1	1	1		1/s
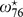	2700	3100	3900	4300	0.3	1/s
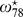	5	5	4	4	0.3	1/s
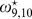	0.27	0.27	0.27	0.27	0.2	1/s
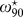	6	6	6	7	0.2	1/s
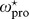	190	200	230	230	0.5	1/s
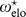	15	18	22	22		aa/s

The values of the overall elongation rate 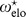 for the four growth conditions 0.7, 1.07, 1.6, and 2.5 dbl/h were obtained from the data in Ref. [27]. These growth conditions have been chosen because, for these conditions, the total tRNA concentrations have been measured as well in Ref. [28]. The relative standard deviations (RSDs) in the sixth column were obtained from the errors of the *in-vitro* rates in Table 1, and from the associated changes in the predicted in-vivo point, see *Methods* section.

### Validation of deduced *in-vivo* rates for translation elongation

**Figure 6:**
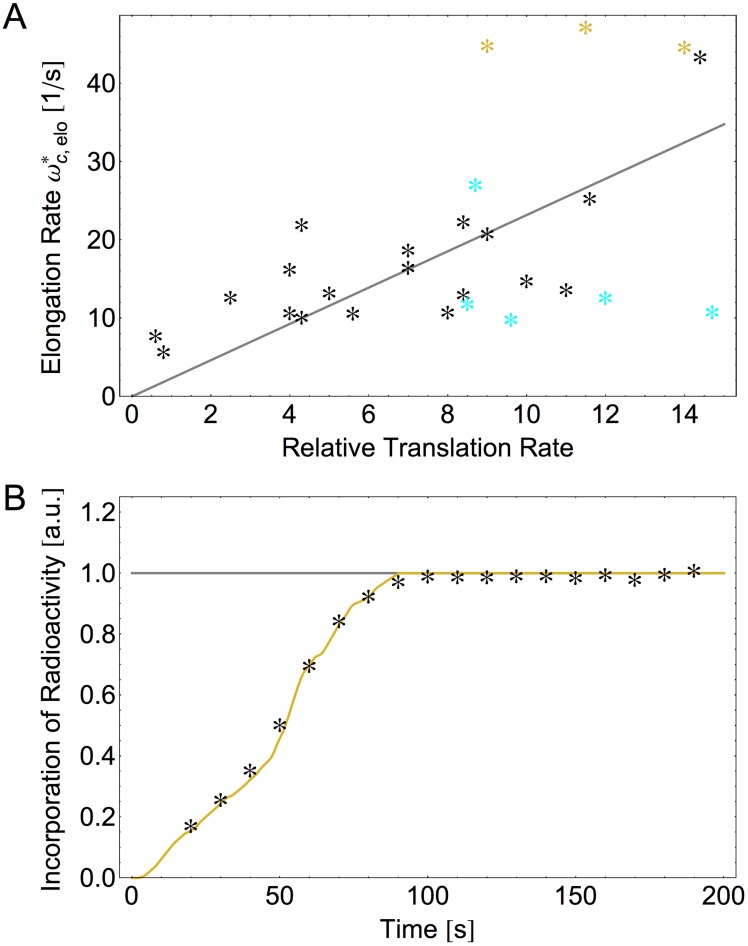
Comparison with *in-vivo* experiments of translation elongation. (A) Codon-specific elongation rates 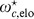 as determined here from the complete set of individual transition rates for *E. coli* at a growth rate of 2.5 dbl/h, see Eq. 21 and Supporting Figure S3, compared to relative translation rates as measured in Ref. [29] for 29 codons; highlighted symbols indicate the codons CGA, CGC, and CGU (orange) as well as the codons UUU, UUC, UUG, UCC, and CCC (cyan), see text. The Pearson correlation coefficient is 0.56 for all codons and 0.73 when the highlighted codons are excluded (linear fit in gray). (B) For the incorporation of radioactively labeled amino acids as a function of time, we find very good agreement between the experimental data in Ref. [30] and the calculated curve (orange) based on the *in-vivo* rates 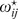 for 0.7 dbl/h in Table 2. For both (A) and (B), our computations do not involve any fit parameter.

Starting from the complete set of individual *in-vivo* rates (Table 2), we computed the codon-specific elongation rates 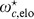 as described in the *Methods* section (Eq. 21, Supporting Figure S3). We then compared the *in-vivo* rates 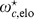 calculated for a growth rate of 2.5 dbl/h to relative translation rates as estimated in Ref. [29] based on the frequencies of the measured +1 frameshifting vs. readthrough of different codons. As shown in Fig. 6A, we obtain reasonable overall agreement between both data sets with a Pearson correlation coefficient of 0.56. The deviations reflect both limitations of our model parametrization and uncertainties in the experimental method. First, the calculated elongation rates for CGA, CGC, and CGU appear to be overestimated. These codons are all read by tRNA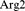, which does not form a Watson-Crick base pair with any of its cognate codons because it carries inosine at the wobble position of its anticodon ICG. The corresponding reductions in the transition rates are not included in the parametrization of our model because we use only two different sets of values for these rates, corresponding to an average over all cognate and over all near-cognate ternary complexes, respectively. Second, for the experimental setup in [29], the UUU, UUC, UUG, UCC, and CCC codons, when located between a preceding CUU codon and a subsequent CXX codon, generate potential slippery sequences, which can lead to 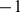 frameshifting events. The latter events were not considered and, thus, not taken into account by [29], which implies that the frameshifting rates were underestimated and the translation rates were overestimated for the respective codons. When we exclude these two particular sets of codons, we obtain an increased correlation coefficient of 0.73 as shown in Fig. 6A. Thus, the deduced values 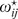 of the individual transition rates *in vivo* lead to a reliable description for the majority of codons.

To further validate these deduced values, we used the computed values 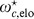 of the codon-specific elongation rates (Supporting Figure S3), to model the time course of protein synthesis measured by [30]. In those experiments, the lacZ gene was expressed in *E. coli* at a growth rate of about 0.7 dbl/h, the cells were exposed to a 10-s pulse of radioactively labeled methionine, and the radioactivity of the synthesized proteins was measured over time. The calculated time course is in excellent agreement with the experimental data (Fig. 6B). Furthermore, varying the values of the internal transition rates leads to significant deviations of the simulation curve from the data (Supporting Figure S4).

Another quantity that can be used to validate the deduced *in-vivo* rates 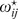 is the missense error frequency arising from the accommodation of near-cognate ternary complexes with incorrect amino acids. The calculated error frequency depends on all individual transitions for the accommodation of a cognate or near-cognate aa-tRNA and, in particular, on the concentrations of cognate and near-cognate ternary complexes whereas it is independent of the concentrations of non-cognates, see Eq. 36 in the *Methods* section. Using the deduced *in-vivo* rates in Table 2 and the ternary complex concentrations as estimated from the measured tRNA concentrations for 0.7 dbl/h [28], we obtain an average missense error frequency of 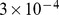 for tRNA^Lys^ misreading codons, in good agreement with the measured value 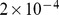 [31].

## Discussion

The theoretical approach described here involves two novel concepts. First, we introduced the kinetic distance to provide a quantitative measure for the similarity of the *in-vitro* and *in-vivo* kinetics. This distance has an intuitive interpretation in terms of the free energy barriers that govern the individual transition rates along the reaction pathways, and provides a genuine metric in the mathematical sense. Second, we constructed a constrained minimization procedure in order to deduce the unknown *in-vivo* values of the individual transition rates from their known *in-vitro* values.

It is instructive to compare our approach with flux control or sensitivity analysis, a widely used method for multistep reaction pathways [3], [35]–[37], which has also been applied to protein synthesis [38]. The latter method explores the *local* vicinity of a given kinetics and describes the *linear* response of the overall flux to small changes in the individual transition rates in terms of flux control or sensitivity coefficients. In contrast, the theoretical approach introduced here is not restricted to the linear response regime but explores the space of transition rates in a *global* manner via an extremum principle (Fig. 2B). Furthermore, both the coordinate transformation from the individual transition rates 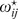 to the single barrier shifts 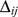 and the constraint arising from the global *in-vivo* property make our approach *highly nonlinear*.

When we applied our computational method to translation elongation by ribosomes, we obtained predictions for the individual *in-vivo* rates 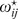 that could be validated by three independent sets of data for codon-dependent translation speeds, codon-specific translation dynamics and missense error frequencies of protein synthesis. In all cases, we found good agreement between theory and experiment without adjusting any fit parameter.

Even for the largest growth condition of 2.5 dbl/h, most of the deduced *in-vivo* rates 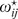 are similar to the measured *in-vitro* rates 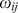 (Fig. 5B) but three *in-vivo* rates are significantly increased compared to their *in-vitro* values: the rejection rate 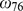 for near-cognates, the dissociation rate 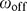 after initial binding, and the recognition rate 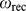 for cognate and near-cognate ternary complexes. The largest difference is found for the rejection rate 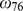, which is increased *in vivo* by a factor of 3.9, while the dissociation rate 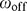 and the recognition rate 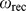 are increased by a factor 3.3 and 2.2, respectively.

For all transition rates of the elongation cycle, we find that the deviations between the *in-vivo* and *in-vitro* rates correspond to relatively small shifts of the corresponding free energy barriers (Fig. 5A). In fact, all single barrier shifts are predicted to be smaller than 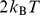. Because the cytosol represents a rather complex buffer, such small changes in the free energy barriers can be easily envisaged, arising, e.g., from changes in the hydrogen bond networks around the ribosome or from changes in the flexibility of some parts of this complex. On the other hand, our results also show that the high-fidelity buffer at 37°C, used here and developed by [25] represents a good approximation to the cytosol as far as the ribosomal kinetics is concerned, in contrast to earlier estimates in Ref. [39].

The free energy barriers considered here could be studied by Molecular Dynamics simulations. The latter method has been recently applied to explore the free energy landscape of tRNA translocation through the ribosome [40], [41]. From such simulations, one can estimate the attempt frequencies for barrier crossing which are difficult to determine by other computational methods. In principle, these simulation techniques could also be used to investigate how the energy landscape changes as one varies the ambient buffer conditions in the simulations.

Even though the predicted shifts of the free energy barriers are relatively small, the associated changes of the transition rates have an interesting consequence for the relative importance of initial selection and proofreading for the error frequency of protein synthesis. For the codon-specific Markov process depicted in Fig. 3, the efficiency of initial selection and proofreading are described by the coefficients 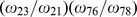 and 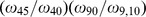, respectively. The *in-vivo* value of the initial selection coefficient is increased by a factor of 7.7 compared to the corresponding *in-vitro* value whereas the proofreading coefficient is increased by a factor of 2.9. The combination of improved initial selection and proofreading leads to a reduction of the *in-vivo* error frequency by a factor of 6.7, a reduction that is primarily achieved by the improved initial selection of the bound ternary complexes.

In the present study, the codon-dependence of the elongation cycle arose from the initial binding rates that depend on the concentrations of cognate, near-cognate, and non-cognate tRNA, because we used the same transition rates along the reaction pathways for all cognate as well as for all near-cognate tRNAs. Thus, the values of the rates 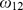, 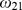, …of the cognate branch represent average values, obtained by averaging over all cognate tRNAs of all codons, and likewise for the internal rates 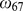, 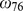, …of the near-cognate branch. *In vitro*, the decoding rates of different cognate codons were observed to be rather similar [14], [25] whereas the GTPase activation rate 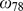 was found to vary between 0.06/s and 1.3/s for different near-cognate codons of tRNA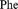 [25]. Likewise, recent *in-vivo* experiments provided evidence that the error frequency on 4 out of 14 near-cognate codons of tRNA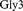 is much higher than on the remaining 10 near-cognate codons [42]. Theoretically, it is straightforward to include codon-specific decoding and processing rates. Experimentally, it is, however, quite challenging to determine these rates *in vitro* for all codons and tRNA species.

Our theory for protein synthesis by ribosomes can be extended in a variety of ways. For example, one could study how the overall elongation rate or the missense error frequency vary with changes in the overall ternary complex composition or as a function of individual ternary complex concentrations. Likewise, one may investigate how changes in internal transition rates arising, e.g., from protein or rRNA mutagenesis, affect the speed and accuracy of translation elongation.

The computational method developed here to deduce the *in-vivo* from the *in-vitro* rates is relatively simple and can be applied, in general, to any multistep process or Markov model, for which one can estimate the *in-vitro* rates. Simple examples are provided by the folding and unfolding of proteins, the catalytic activity of enzymes with one active site, or the motility of molecular motors. More complex examples are transcription by RNA polymerase, protein refolding by chaperones, or protein degradation by proteases. Our method can also be applied to the large number of biochemical processes that have been studied by flux control or sensitivity analysis. Furthermore, the similarity measure provided by the kinetic distance could be useful in the context of systems biology, where the importance of detailed kinetics has been recently emphasized [43]. One important target in systems biology is to standardize the experimental data for such networks. Using the kinetic distance introduced here, one could, in fact, compare the kinetic data obtained by different groups in a systematic and quantitative manner.

## Methods

### Codon-specific accommodation times

Using the general theory of stochastic processes [44], [45], we derived explicit expressions for important dynamical quantities of the translation elongation cycle (Fig. 3) in terms of the individual transition rates. These quantities include the codon-specific accommodation times, i.e., the times that the ribosome needs to fully accommodate a cognate or near-cognate tRNA and, thus, to move from state 0 to state 5 or state 10 for the Markov process in Fig. 3. A straightforward but somewhat tedious computation leads to explicit, analytical expressions for these time scales in terms of the individual transition rates 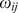. These expressions can be decomposed into four different dwell times according to
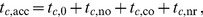
(11)a decomposition that directly reflects the state space of the Markov process in Fig. 3 and has the following intuitive interpretation.

The first dwell time 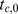 represents the total time that the ribosome spends in state 0 during one complete elongation cycle at codon 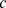. Because of the different dissociation and backward transitions, the ribosome typically visits the state 0 several times before it is fully accommodated in the states 5 or 10, see Fig. 3. The second dwell time 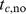 in Eq. 11 corresponds to the total time that the ribosome binds a non-cognate ternary complex and, thus, dwells in state 11 during one complete elongation cycle at codon 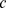. The third dwell time 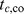 corresponds to the total time that the ribosome spends in the intermediate states 1, 2, 3, and 4 of the cognate branch during one complete elongation cycle at codon 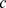. Finally, the fourth dwell time 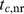 in Eq. 11 represents the total time that the ribosome spends in the intermediate states 6, 7, 8, and 9 of the near-cognate branch.

These four dwell times can be expressed in a particularly compact and transparent manner if one uses the transition probabilities
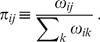
(12)


The dwell time 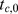, which the ribosome spends in state 0 during one complete elongation cycle at codon 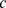, then has the form
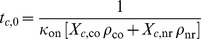
(13)and, thus, depends on the concentrations 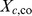 and 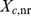 of free cognate and near-cognate ternary complexes as well as on the dimensionless, concentration-independent ratios
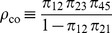
(14)and



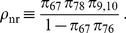
(15)The second dwell time 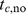 for state 11 with a bound non-cognate ternary complex is given by the expression
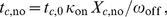
(16)and is, thus, proportional to the concentration 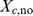 of free non-cognate ternary complexes.

The third dwell time 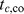, which represents the sum of all dwell times for the intermediate states 1, 2, 3, and 4 of the cognate branch, can be written as
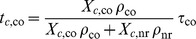
(17)with the concentration-independent time scale
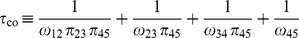
(18)that depends only on transitions that emanate from the intermediate states of the cognate branch. Likewise, the fourth dwell time 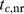 for the intermediate states 6, 7, 8, and 9 of the non-cognate branch has the form
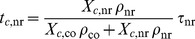
(19)with the concentration-independent time scale
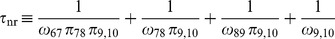
(20)that depends only on transitions that emanate from the intermediate states of the near-cognate branch.

### Codon-specific and overall elongation rates

The expression for the codon-specific accommodation time 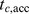 as given by Eq. 11 involves all individual rates 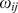 apart from the processing rate 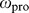. When we add the processing time 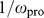, we obtain the codon-specific elongation time 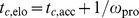 which the ribosome needs to complete a full elongation cycle at a certain codon 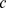. The codon-specific elongation rates are then given by
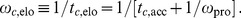
(21)


One important global property of protein synthesis is the average speed of the ribosomes, which defines the overall elongation rate 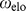. The inverse of the overall elongation rate is equal to the average elongation time 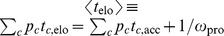, which is obtained by averaging the codon-specific elongation times 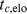 over all codons 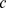 using the codon usage 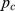. For each codon 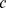, the quantity 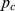 represents the probability that the ribosome encounters this codon. These probabilities are normalized and satisfy 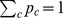.

For the *in-vitro* assay, the relation between the overall elongation rate 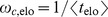 and the codon-specific accommodation times 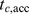 was rewritten in the form
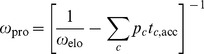
(22)and then used to calculate the processing rate 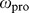 from the measured value of the overall elongation rate 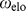 and the measured values of the individual rates 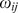, which determine the codon-specific accommodation times 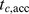.

*In vivo*, the overall elongation rate 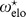 is given by the analogous expression
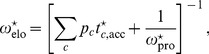
(23)where the codon-specific accommodation times 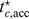 follow from the same expression as in Eq. 11 but with the *in-vitro* values 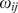 replaced by the *in-vivo* values 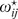. When we insert the known *in-vivo* value 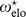 of the overall elongation rate into Eq. 23, we obtain a constraint on the (unknown) *in-vivo* values 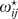 of the individual transition rates. This constraint can be expressed in terms of the single barrier shifts 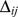 when we replace 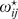 in Eq. 23 by 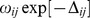, see Eq. 2.

### *In-vitro* values of individual transition rates

All *in-vitro* values of the individual transition rates as given in Table 1 have been obtained for the high-fidelity buffer as developed in [12], [25], and [34]. Most of these values are based on previous measurements as explained in the following paragraph. In addition, we also performed new experiments to measure the overall elongation rate 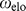, both at 20°C and at 37°C, see Supporting Figure S1, as well as the individual rates 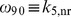 and 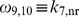 at 20°C, see Supporting Figure S2, using the experimental protocols described previously [34], [46], [47].

The *in-vitro* value 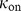 of the association rate constant was previously measured at 20°C [12]. Its value at 37°C was obtained assuming an Arrhenius temperature dependence and using the previously determined activation energy of 2.4 kcal/mol for initial binding [10]. The dissociation rate 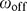 at 20°C was taken from [12]. The decoding rates at 20°C were obtained by averaging over previously published values as measured for different codons of tRNA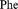. In particular, we averaged the rates as given in Table 1 of [25] for cognate as well as for near-cognate codons to obtain the rates 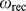, 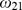, 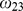, 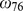, and 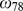. The rate 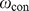 has not been measured but estimated under the assumption that it is not rate-limiting. The rate 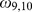 at 37°C was reported previously and was used to determine the rate 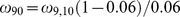, i.e., using an error frequency of 0.06 for the proofreading step [34]. The rate 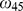 has been measured both for 20°C and for 37°C [25], [34]. The rate 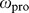 was calculated for both temperatures from the measured values of the overall elongation rate 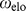 via Eq. 22.

Finally, we assumed an Arrhenius temperature dependence to estimate some of the *in-vitro* rates at 37°C from their values as measured at 20°C. These estimates are based on the following considerations. We start from Eq. 4 for the transition rates and use the decomposition 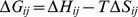 of the activation free energy 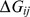 into the activation enthalpy 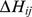 and the activation entropy 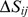, which leads to
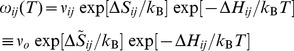
(24)where the last expression involves the attempt frequency 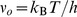 as obtained from transition-state theory [2]. In this way, any state-dependence of the attempt frequency 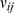 has been absorbed into the activation entropies 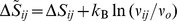. If one plots the logarithms 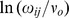 of the measured rates 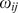 as a function of the inverse temperature 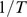 (conventional Arrhenius plots), one finds linear relationships [10], [13], [48], [49], which imply that the two unknown parameters in Eq. 24, 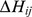 and 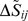, do not depend on temperature over the experimentally studied temperature range. However, the activation entropies 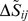 as obtained from the behavior of 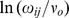 for small 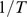 vary significantly with the ribosomal states 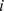 and 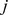 [10], [13], [48], [49]. Possible molecular mechanisms for this variation have been recently discussed based on atomistic molecular dynamics simulations [50].

Using the expression in Eq. 24 with 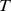-independent enthalpies 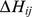 and entropies 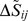, we now consider the ratios 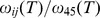 at the two temperatures of interest, 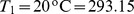
K and 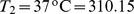
K. We take the accommodation rate 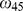 as a reference rate because the value of this rate has been measured at both temperatures. For each individual transition, we then obtain two equations, corresponding to the two temperatures 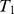 and 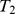, which can be combined to eliminate the enthalpy 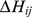. As a result, we obtain the relation
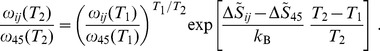
(25)


At present, the entropy differences 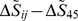 are difficult to estimate for all individual transitions from the available experimental data. However, these differences are multiplied by the relative temperature difference 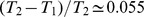 which is rather small. Therefore, we used the approximate relation
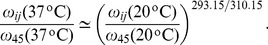
(26)to estimate the values of the rates 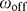, 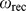, 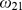, 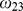, 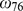, 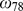, 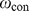, and 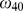 at 37°C (Table 1) from the measured values of 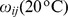, 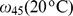, and 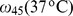.

### *In-vivo* values of association rates

The overall elongation rate 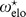 as given by Eq. 23 also depends on the association rates for initial binding, which are proportional to the pseudo-first-order rate constant 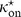 and to the concentrations 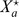 of the ternary complexes as in Eq. 9. Therefore, in order to use Eq. 23 for the process *in vivo*, we had to estimate the corresponding values 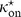 and the ternary complex concentrations 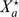 in the cell.

The diffusion of ternary complexes and, thus, their binding to ribosomes is slowed down *in vivo* by molecular crowding. The time it takes a ternary complex to find a single ribosome depends on the cell volume, the diffusion constant of the ternary complex, and the ribosome size [51]. Using the diffusion constant of 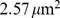/s [52], [53] for a ternary complex in the cytosol, we found that the *in-vivo* value 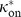 of the bimolecular association rate constant is about 54% of the *in-vitro* value 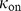, compare Table 1 and Table 2.

For the *in-vivo* concentrations 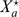 of the ternary complexes, we used the values of the tRNA concentrations as measured by [28] in *E. coli* for the growth conditions of 0.7, 1.07, 1.6, and 2.5 dbl/h. In the latter study, the authors determined the concentrations 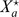 of all 43 elongator tRNA species 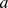. These concentrations are then combined, for each codon 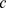, into the concentrations 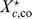, 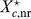, and 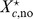 of cognate, near-cognate, and non-cognate ternary complexes within the cell. Thus, for each codon 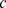, we started from the corresponding row in Fig. 4, and added all concentrations 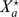 up that correspond to green (cognate), yellow (near-cognate), and purple (non-cognate) tRNA species, respectively.

### Uncertainty of predicted *in-vivo* rates

To estimate the uncertainty of the predicted *in-vivo* rates 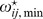, we first simplify the notation. In this section, the internal transitions with distinct transition rates will be distinguished by the subscript 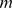 with 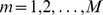. Thus, we now use the short-hand notation 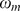, 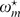, and 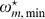 for the *in-vitro* rates 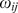 of a certain assay, for the unknown *in-vivo* rates 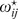, and for the predicted *in-vivo* rates 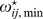, respectively. For ribosome elongation as described by the Markov process in Fig. 3, we distinguish 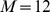 internal transitions.

The inaccuracy or error of the *in-vitro* rates can be described by
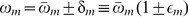
(27)with the absolute error 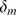 and the relative error
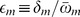
(28)of the *in-vitro* rate 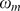. Both the average values 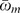 and the absolute errors 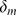 are estimated from the experimental data for the *in-vitro* assay under consideration.

When we apply the minimization procedure to the average values 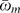 of the *in-vitro* rates, we use the coordinates
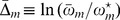
(29)for the multi-dimensional barrier space. We then determine the point 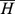 that is located on the hypersurface defined by Eq. 8 and depicted in Fig. 2B and has the shortest distance from the origin of the 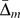-coordinates. The coordinate values 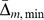 of the point 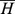 then lead to the predicted values 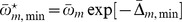 for the *in-vivo* rates.

In order to estimate the uncertainty of these predictions, it is useful to consider an auxiliary ensemble of fictitious *in-vitro* assays that is constructed ‘around’ the given assay as follows. For each transition 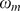, we introduce the binary variable 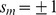. The 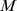 binary variables 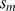 can assume 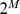 different ‘configurations’ 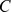 as described by the different 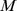-tuples
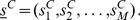
(30)


Each of these configurations defines a fictitious *in-vitro* assay, again denoted by 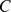, with transition rates
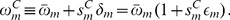
(31)


The rates of assay 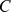 define the coordinates
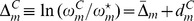
(32)for the multi-dimensional barrier space with
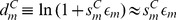
(33)where the asymptotic equality applies to the limit of small relative errors 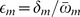 of the *in-vitro* rates. Therefore, if the origin of the multi-dimensional barrier space is defined by the coordinates 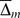 in Eq. 29, corresponding to the average *in-vitro* rates 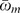, the ensemble of the fictitious assays 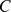 forms the corners of a multi-dimensional ‘error polyhedron’ around this origin. For each corner, again labeled by 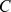, we can apply our minimization procedure and minimize the kinetic distance of the point 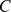 from the hypersurface as defined by Eq. 8 and depicted in Fig. 2B. The point 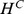 on the hypersurface with the shortest distance from the corner 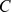 has the coordinates 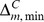.

The variations in the coordinate values 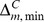 of the predicted *in-vivo* point as obtained for different corners 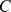 can be used to obtain an estimate for the absolute error 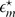 of these coordinate values. We then write the coordinate values of the predicted *in-vivo* point in the form
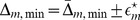
(34)where the values 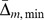 correspond to the average *in-vitro* rates 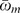. The predicted *in-vivo* rates are now given by



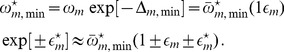
(35)Therefore the relative error of the predicted *in-vivo* rates 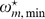 reflects both the relative error 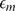 of the *in-vitro* rates 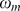 (Eq. 27) and the absolute error 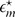 of the coordinate values 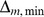 for the predicted *in-vivo* point (Eq. 34).

The Markov process for ribosome elongation considered here, see Fig. 3, involves 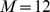 distinct transition rates, which implies that the corresponding barrier space has 12 dimensions. We first determined the coordinate values 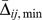 of the *in-vivo* point as predicted from the average value 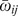 of the *in-vitro* rates. The largest coordinate values 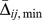 of the predicted *in-vivo* point were found for the three transition rates 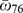, 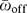, and 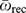 (Fig. 5B). We then focused on the errors of these three *in-vitro* rates, which define 8 corners of the ‘error polyhedron’ around the origin of the 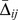-coordinates. For each of these corners 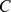, we determined the closest point on the hypersurface and the coordinate values 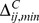 of this point. We then estimated the absolute error 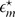 of the coordinate values 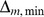 from the largest and smallest values of 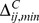 as obtained for different corners 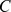. The errors 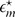 were finally used, together with the relative errors 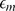 of the measured *in-vitro* rates, to determine the relative standard deviations (RSDs) of the predicted *in-vivo* rates as displayed in Table 2.

### Missense error frequency

Consider a certain tRNA species 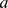 and a codon 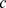 that is near-cognate to 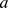. The missense error frequency for misreading the codon 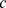 by the tRNA species 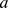 is equal to the probability 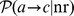 that 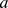 is fully accommodated at 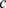. For the multistep process considered here, this probability is given by
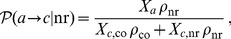
(36)which depends on the concentration 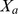 of the near-cognate ternary complex species 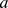, on the concentrations 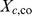 and 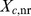 of all cognate and near-cognate ternary complexes as well as on the concentration-independent ratios 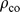 and 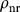 as given by Eqs. 14 and 15.

The experimental study in [31] determined the error frequency for all codons that are near-cognate to 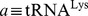. The average error frequency for misreading one of these codons is then obtained from
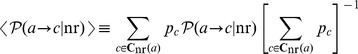
(37)where the set 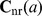 contains all codons 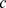 that are near-cognate to 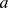 and 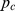 denotes the codon usage as before.

## Supporting Information

Figure S1 **Overall elongation rate as measured for a model protein** ***in vitro***. Kinetics of CspA translation *in vitro* at different temperatures. CspA mRNA, which codes for a 70 aa-long protein from *E. coli*, was prepared by T7 RNA-polymerase transcription. Ribosomes were synchronized by forming an initiation complex consisting of 70S ribosomes, CspA mRNA and a fluorescence derivative of initiator tRNA^fMet^ carrying BodipyFL at the *α*-amino group of Met in the presence of initiation factors (IF1, IF2, and IF3) and GTP. Translation was carried out in a fully reconstituted translation system by adding initiation complexes (15 nM) to a mixture of EF-Tu–GTP–aminoacyl-tRNA (40 µM aminoacyl-tRNA, 100 µM EF-Tu in total), EF-G (3 µM), GTP (2 mM), phosphoenol pyruvate (6 mM), and pyruvate kinase (0.1 mg/ml) in HiFi buffer (50 mM Tris-HCl, pH 7.5, 30 mM KCl, 70 mM NH_4_Cl, 3.5 mM free MgCl_2_, 0.5 mM spermidine, and 8 mM putrescine) at the indicated temperatures [46]. In the absence of translation termination and ribosome recycling factors, translation was limited to a single round, i.e. at most one molecule of CspA was synthesized per ribosome. The reactions were stopped at the indicated time intervals and translation products separated on 16.5% Tris-Tricine-PAGE and visualized by the fluorescent reporter BODIPY-Fl at the N-terminus of the peptides [47] (left panels). The intensity of the full length product was quantified with ImageJ (right panel, circles). Average translation rates per codon, which depend on the elongation rates only, were determined by exponential fitting (fits in graphs of the right panel).
(TIF)

Figure S2
***In-vitro*** **rates as measured for near-cognate accommodation and rejection after proofreading.** *In-vitro* values of the rates 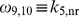 and 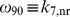 for near-cognate accommodation and rejection after proofreading at 20°C as determined by the experimental protocol described previously in Ref. [34]. The formation of f[^3^H]Met[^14^C]Phe was monitored under multiple-turnover conditions using initiation complexes 70S–mRNA(AUGCUC)–f[^3^H]Met-tRNA^fMet^ (0.14 μM) and varying concentrations of the ternary complex EF-Tu–GTP–[^14^C]Phe-tRNA^Phe^, which is near-cognate to the CUC codon. For each concentration of the ternary complex, the rates were determined from the linear slopes of the time courses. From the hyperbolic dependence of the concentration dependence of 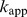, we calculated 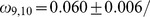
s and 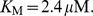 Using the previously measured efficiency 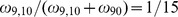 of the proofreading step [25], we then obtained the value 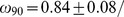
s for near-cognate rejection after proofreading.(TIF)

Figure S3
**Codon-specific elongation rates** ***in vitro*** **and** ***in vivo*** **.** Codon-specific elongation rates 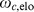 in units of amino acids per second as calculated from Eq. 17, see *Methods* section in the main text, using the decomposition of the codon-specific elongation times in Eq. 7 and the complete sets of individual transition rates: (A) *In-vitro* values 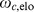 for the high-fidelity buffer at 37°C, obtained from the individual rates in Table 1; (B, C) *In-vivo* values 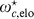 for *E. coli* at growth conditions of (B) 0.7 dbl/h and (C) 2.5 dbl/h, calculated from the individual rates in Table 2.(TIF)

Figure S4
**Incorporation of radioactively labeled amino acids for different dissociation rates.** Experimental data (black stars) for the incorporation of radioactively labeled amino acids at a growth rate of 0.7 dbl/h [30] and simulation curves obtained for five different values of the initial dissociation rate 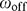. The orange simulation curve in the middle corresponds to 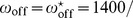
s, see Table 2. This value has been obtained from the minimization of the kinetic distance and provides an excellent fit to the data. The red, blue, green, and black curves have been obtained for simulations with 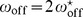, 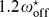, 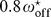, and 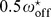, respectively. Thus, changing the value of 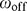 by 20% leads to a significant deviation of the simulation curve from the experimental data.(TIF)
