# Supplementary figures and images for: Deducing the Kinetics of Protein Synthesis In Vivo from the Transition Rates Measured In Vitro
Source: PLoS Comput Biol. 2014 Oct 30;10(10):e1003909. doi: 10.1371/journal.pcbi.1003909 (PMC4214572; doi:10.1371/journal.pcbi.1003909)

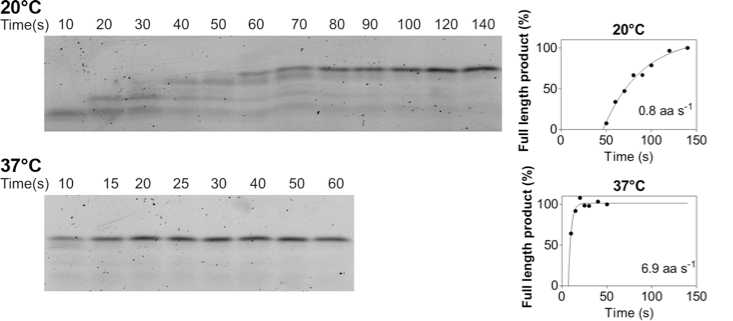

Supplement: Figure S1 — Overall elongation rate as measured for a model protein in vitro. Kinetics of CspA translation in vitro at different temperatures. CspA mRNA, which codes for a 70 aa-long protein from E. coli, was prepared by T7 RNA-polymerase transcription. Ribosomes were synchronized by forming an initiation complex consisting of 70S ribosomes, CspA mRNA and a fluorescence derivative of initiator tRNAfMet carrying BodipyFL at the α-amino group of Met in the presence of initiation factors (IF1, IF2, and IF3) and GTP. Translation was carried out in a fully reconstituted translation system by adding initiation complexes (15 nM) to a mixture of EF-Tu–GTP–aminoacyl-tRNA (40 µM aminoacyl-tRNA, 100 µM EF-Tu in total), EF-G (3 µM), GTP (2 mM), phosphoenol pyruvate (6 mM), and pyruvate kinase (0.1 mg/ml) in HiFi buffer (50 mM Tris-HCl, pH 7.5, 30 mM KCl, 70 mM NH4Cl, 3.5 mM free MgCl2, 0.5 mM spermidine, and 8 mM putrescine) at the indicated temperatures [46]. In the absence of translation termination and ribosome recycling factors, translation was limited to a single round, i.e. at most one molecule of CspA was synthesized per ribosome. The reactions were stopped at the indicated time intervals and translation products separated on 16.5% Tris-Tricine-PAGE and visualized by the fluorescent reporter BODIPY-Fl at the N-terminus of the peptides [47] (left panels). The intensity of the full length product was quantified with ImageJ (right panel, circles). Average translation rates per codon, which depend on the elongation rates only, were determined by exponential fitting (fits in graphs of the right panel). (TIF) [file pcbi.1003909.s001.tif]

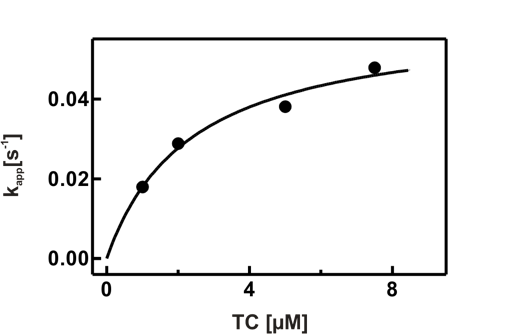

Supplement: Figure S2 — In-vitro rates as measured for near-cognate accommodation and rejection after proofreading. In-vitro values of the rates and for near-cognate accommodation and rejection after proofreading at 20°C as determined by the experimental protocol described previously in Ref. [34]. The formation of f[3H]Met[14C]Phe was monitored under multiple-turnover conditions using initiation complexes 70S–mRNA(AUGCUC)–f[3H]Met-tRNAfMet (0.14 μM) and varying concentrations of the ternary complex EF-Tu–GTP–[14C]Phe-tRNAPhe, which is near-cognate to the CUC codon. For each concentration of the ternary complex, the rates were determined from the linear slopes of the time courses. From the hyperbolic dependence of the concentration dependence of , we calculated s and Using the previously measured efficiency of the proofreading step [25], we then obtained the value s for near-cognate rejection after proofreading. (TIF) [file pcbi.1003909.s002.tif]

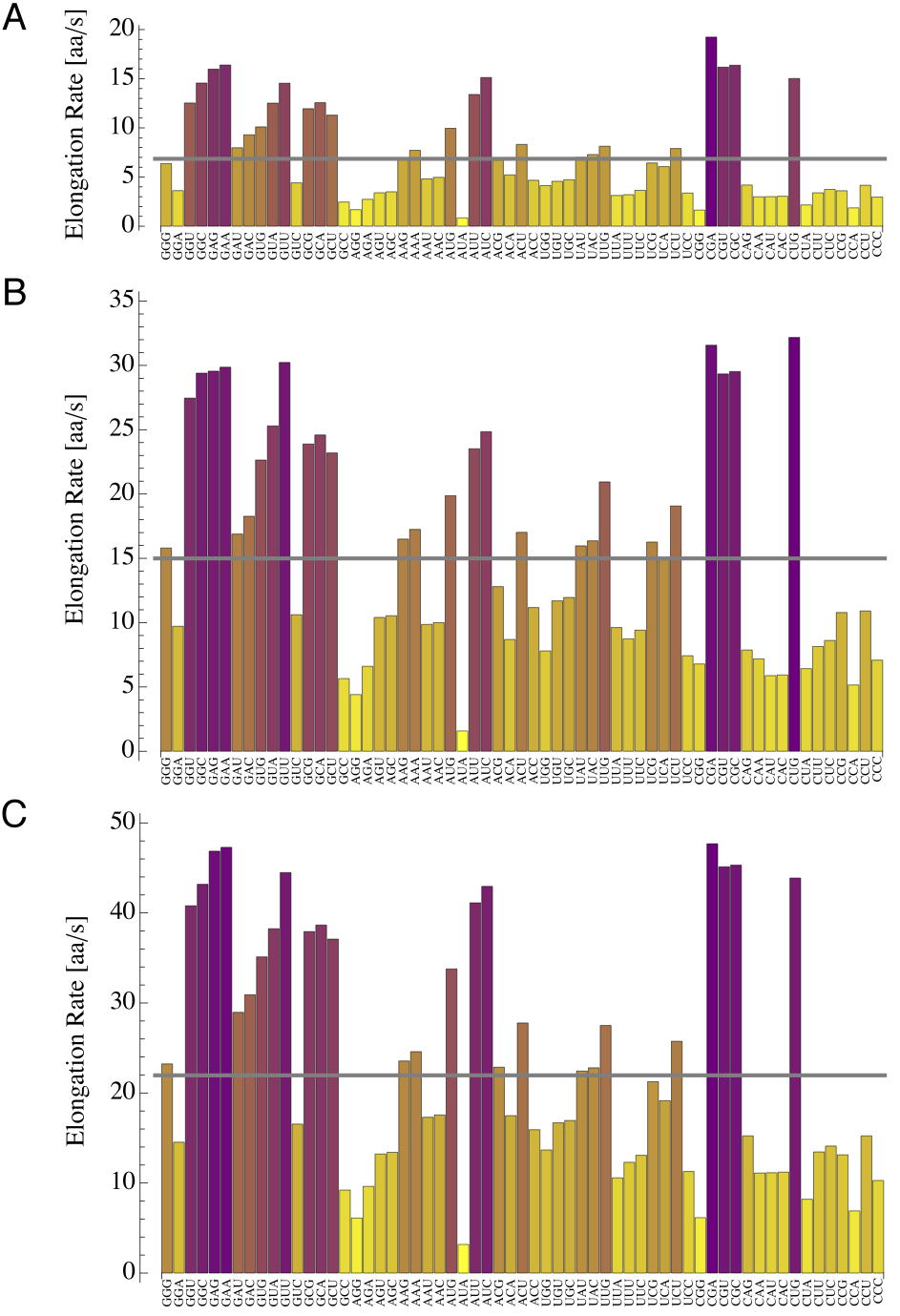

Supplement: Figure S3 — Codon-specific elongation rates in vitro and in vivo . Codon-specific elongation rates in units of amino acids per second as calculated from Eq. 17, see Methods section in the main text, using the decomposition of the codon-specific elongation times in Eq. 7 and the complete sets of individual transition rates: (A) In-vitro values for the high-fidelity buffer at 37°C, obtained from the individual rates in Table 1; (B, C) In-vivo values for E. coli at growth conditions of (B) 0.7 dbl/h and (C) 2.5 dbl/h, calculated from the individual rates in Table 2. (TIF) [file pcbi.1003909.s003.tif]

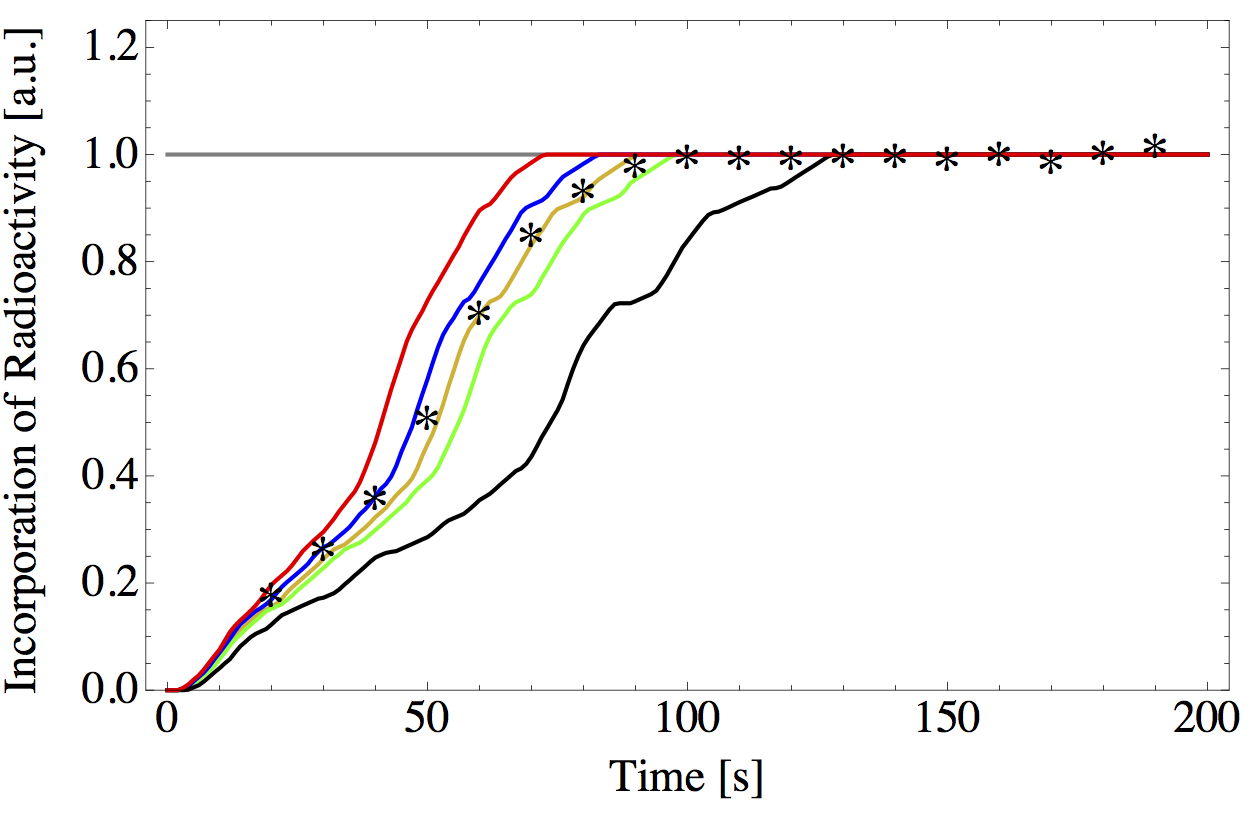

Supplement: Figure S4 — Incorporation of radioactively labeled amino acids for different dissociation rates. Experimental data (black stars) for the incorporation of radioactively labeled amino acids at a growth rate of 0.7 dbl/h [30] and simulation curves obtained for five different values of the initial dissociation rate . The orange simulation curve in the middle corresponds to s, see Table 2. This value has been obtained from the minimization of the kinetic distance and provides an excellent fit to the data. The red, blue, green, and black curves have been obtained for simulations with , , , and , respectively. Thus, changing the value of by 20% leads to a significant deviation of the simulation curve from the experimental data. (TIF) [file pcbi.1003909.s004.tif]
